# Multilevel psychometric properties of the AHRQ hospital survey on patient safety culture

**DOI:** 10.1186/1472-6963-10-199

**Published:** 2010-07-08

**Authors:** Joann S Sorra, Naomi Dyer

**Affiliations:** 1 Westat, Rockville, MD, USA

## Abstract

**Background:**

The Agency for Healthcare Research and Quality (AHRQ) *Hospital Survey on Patient Safety Culture *was designed to assess staff views on patient safety culture in hospital settings. The purpose of this study was to examine the multilevel psychometric properties of the survey.

**Methods:**

Survey data from 331 U.S. hospitals with 2,267 hospital units and 50,513 respondents were analyzed to examine the psychometric properties of the survey's items and composites. Item factor loadings, intraclass correlations (ICCs), design effects, internal consistency reliabilities, and multilevel confirmatory factor analyses (MCFA) were examined as well as intercorrelations among the survey's composites.

**Results:**

Psychometric analyses confirmed the multilevel nature of the data at the individual, unit and hospital levels of analysis. Results provided overall evidence supporting the 12 dimensions and 42 items included in the AHRQ *Hospital Survey on Patient Safety Culture *as having acceptable psychometric properties at all levels of analysis, with a few exceptions. The Staffing composite fell slightly below cutoffs in a number of areas, but is conceptually important given its impact on patient safety. In addition, one hospital-level model fit indicator for the Supervisor/Manager Expectations & Actions Promoting Patient Safety composite was low (CFI = .82), but all other psychometrics for this scale were good. Average dimension intercorrelations were moderate at .42 at the individual level, .50 at the unit level, and .56 at the hospital level.

**Conclusions:**

Psychometric analyses conducted on a very large database of hospitals provided overall support for the patient safety culture dimensions and items included in the AHRQ *Hospital Survey on Patient Safety Culture*. The survey's items and dimensions overall are psychometrically sound at the individual, unit, and hospital levels of analysis and can be used by researchers and hospitals interested in assessing patient safety culture. Further research is needed to study the criterion-related validity of the survey by analysing the relationship between patient safety culture and patient outcomes and studying how to improve patient safety culture.

## Background

Patient safety culture, a specific aspect of an organization's overall culture, has received growing attention as a focus on patient safety in healthcare organizations has become an international priority. The concept of safety culture emerged from research focused on safety and accident prevention in high reliability, error-critical industries such as aviation, chemical and nuclear power plants, and manufacturing [1-5]. Establishing a culture of safety has been determined to be a key element of high reliability organizations [6,7]. The concept of safety culture is increasingly seen as central to the understanding of patient safety in healthcare settings. Patient safety culture refers to management and staff values, beliefs, and norms about what is important in a health care organization, how organization members are expected to behave, what attitudes and actions are appropriate and inappropriate, and what processes and procedures are rewarded and punished with regard to patient safety.

The examination of safety culture has also highlighted the importance of understanding the nature of human error and methods of error prevention, including the use of nonpunitive, "just" error reporting systems to identify system anomalies and vulnerabilities [8-10]. However, the actual implementation of these error management and reporting practices in healthcare organizations often runs into numerous barriers. Some of these barriers are fear of reprisals and lack of feedback after errors are reported [11], not fully distributing and working with error reports [12], and a perceived lack of resulting system changes [13].

To make improvements in patient safety, it is important for healthcare organizations to assess the status of their existing culture of patient safety and determine areas of priority to target for improvement [14]. While a number of quantitative organizational culture survey instruments have been developed and used in health care settings [15], they tend to measure a wide range of general cultural dimensions without a specific focus on patient safety. Therefore, a number of surveys specifically assessing patient safety culture have emerged [16,17] and reviews comparing some of these surveys have been published [18-20]. Each of the surveys measures somewhat different dimensions of patient safety culture and it is beneficial for researchers and hospital administrators to have a broad variety of tools from which to choose to best accommodate their purposes for patient safety culture measurement.

The primary goal of the present study was to assess the psychometric properties and dimensionality of the Agency for Healthcare Research and Quality (AHRQ) *Hospital Survey on Patient Safety Culture *(Hospital SOPS)[21]. Another goal was to conduct analyses to determine whether the survey's constructs are useful for assessing patient safety culture at multiple levels: at the individual, department or unit, and hospital levels. Patient safety culture survey data from a large comparative database of U.S. hospitals was used to assess the psychometric properties of the AHRQ Hospital SOPS Survey.

## Methods

### Development of the Survey and Comparative Database

The AHRQ Hospital SOPS was developed by researchers at Westat under an AHRQ contract [22]. To develop the survey, a literature review was conducted in the areas of safety management and accidents; organizational and safety climate and culture; medical error and error reporting; and patient safety. Existing safety climate and culture instruments were also examined. Then, key dimensions of patient safety culture were identified and survey items were developed. The draft survey was cognitively tested and reviewed by researchers and hospital administrators for further input. The survey was pilot tested in 2003 in 21 hospitals across six states in the U.S. The pilot data from 1,437 respondents was analyzed examining item response variability, reliability, and the exploratory and confirmatory individual-level factor structure of the safety culture dimensions [22]. Based on the pilot study's psychometric results, items were dropped, resulting in sets of items comprising independent and reliable safety culture dimensions (reliabilities ranged from .63 to .84). The survey was finalized and made available by AHRQ in November 2004.

In 2006, AHRQ funded the development of a comparative database to serve as a central repository for data from U.S. hospitals that had administered the Hospital SOPS. A call for data submission was made public and 382 hospitals voluntarily submitted data on the survey representing a total of over 100,000 hospital staff respondents. A Comparative Database Report [23] was released in 2007 presenting the database results on the survey's items and composites. Because the survey development pilot test was done on a very limited number of hospitals, in the present study we used data from the larger 2007 database of hospitals to examine the psychometric properties of the survey. Hospitals submitting data to the Comparative Database sign a data use agreement that allows their de-identified data to be made available for health care research purposes so no additional permissions from the hospitals were required for this analysis.

### Analysis Dataset

To examine the psychometric properties of the Hospital SOPS at multiple levels of analysis, it was necessary to refine the 2007 database data to keep only those hospitals and units that met certain criteria. Hospitals were dropped from the analysis dataset because: 1) they did not administer the entire survey; 2) they did not ask the work unit question; or 3) they only had one unit respond. Units within hospitals were dropped from the analysis data set if 1) there were fewer than 3 respondents from the unit, or 2) if the unit was identified as "Other" or "Many different work units" since individuals in these categories do not belong to the same unit and therefore should not be grouped together for analysis purposes. Based on these criteria, a total of 51 hospitals, 1,276 units, and 58,108 respondents were dropped from the 2007 database to create the analysis dataset.

### Sample & Response Statistics

The final analysis dataset consisted of 331 hospitals with 2,267 units, and 50,513 hospital staff respondents. Response rates were calculated from self-reported numbers provided by the hospitals indicating how many staff were asked to participate in the survey across the hospital. Approximately 77% of the hospitals indicated they surveyed all staff, or a sample of all staff, from all departments. The remaining hospitals surveyed a combination of selected staff and/or selected departments. The average response rate for these 331 hospitals (prior to deletion of units) was 55% (range: 6% to 100%), with an average of 289 respondents per hospital (range: 11 to 3,684). Hospitals mainly administered the survey in paper form (58%), with some using web surveys (23%) and others using both paper and web surveys (19%). Table 1 shows the distribution of the hospitals by bed size. The analysis hospitals were primarily non-teaching (77%) and non-government owned (71%).

**Table 1:** Distribution of Analysis Dataset Hospitals by Bed Size

	Hospitals	
	
Bed Size	Number	Percent
6 - 49 beds	124	37%

50-199 beds	117	35%

200-399 beds	62	19%

400 or more beds	28	8%

Total	331	100%

Tables 2 and 3 show the staff positions and work areas of the dataset respondents. Table 2 shows that the largest percentage of respondents were nurses (45% RN, LVN, or LPN), with only 5% physicians/residents/physician assistants/nurse practitioners.

**Table 2:** Distribution of Respondents by Staff Position

Staff Position	Respondents	
	
	Number	Percent
Registered Nurse (RN) or Licensed Vocational Nurse (LVN)/Licensed Practical Nurse (LPN)	21,830	45%

Other	5,292	11%

Technician (EKG, Lab, Radiology)	7,787	16%

Administration/Management	1,715	4%

Unit Assistant/Clerk/Secretary	3,186	7%

Patient Care Asst/Hospital Aide/Care Partner	2,776	6%

Attending/Staff Physician, Resident Physician/Physician in Training, or Physician Assistant (PA)/Nurse Practitioner (NP)	2,677	5%

Therapists (Respiratory, Physical, Occupational or Speech)	2,136	4%

Pharmacist	1,215	2%

Dietician	120	< 1%

TOTAL	48,734	100%

Missing: Did not answer or were not asked the question	1,779	

Overall total	50,513	

**Table 3:** Distribution of Respondents by Work Area/Unit

Work Area/Unit	Respondents				
	
	Number	Percent	Average	Min	Max
Surgery	8,679	17%	34	3	272

Medicine	7,722	15%	28	3	554

Intensive care unit (any type)	5,612	11%	33	3	323

Radiology	5,292	10%	19	3	218

Emergency	4,897	10%	20	3	153

Laboratory	4,673	9%	17	3	164

Rehabilitation	3,954	8%	17	3	140

Obstetrics	3,543	7%	23	3	195

Pharmacy	2,321	5%	12	3	100

Psychiatry/mental health	1,648	3%	20	3	111

Pediatrics	1,530	3%	20	3	102

Anesthesiology	642	1%	16	3	88

**TOTAL**	50,513	100%	22	3	554

As shown in Table 3, the largest percentage of respondents was from Surgery (17%) or Medicine (15%). Table 3 also shows the average, minimum, and maximum number of respondents from each work area. Most respondents (86%) had direct interaction with patients.

### Measures

The AHRQ Hospital SOPS assesses hospital staff opinions about patient safety issues, medical error and event reporting and includes 42 items that measure 12 dimensions or composites of patient safety culture. Most items use 5-point response scales of agreement ("Strongly disagree" to "Strongly agree") or frequency ("Never" to "Always"). Table 4 provides descriptions of the patient safety culture dimensions and the number of survey items measuring each dimension.

**Table 4:** Patient Safety Culture Composites

Patient Safety Culture Composite	**Definition: The extent to which...**.	Number of Survey Items
1. Communication openness	Staff will freely speak up if they see something that may negatively affect patient care, and feel free to question those with more authority	3

2. Feedback & communication about error	Staff are informed about errors that happen, given feedback about changes put into place based on event reports, and discuss ways to prevent errors	3

3. Frequency of events reported	Mistakes of the following types are reported: 1) mistakes caught and corrected before affecting the patient, 2) mistakes with no potential to harm the patient, and 3) mistakes that could harm the patient, but do not	3

4. Handoffs & transitions	Important patient care information is transferred across hospital units and during shift changes	4

5. Management support for patient safety	Hospital management provides a work climate that promotes patient safety and shows that patient safety is a top priority	3

6. Nonpunitive response to error	Staff feel that their mistakes are not held against them, and mistakes are not kept in their personnel file	3

7. Organizational learning--Continuous improvement	Mistakes have led to positive changes and changes are evaluated for their effectiveness	3

8. Overall perceptions of patient safety	Procedures and systems are good at preventing errors and there is a lack of patient safety problems	4

9. Staffing	There are enough staff to handle the workload and work hours are appropriate to provide the best care for patients	4

10. Supervisor/manager expectations and actions promoting safety	Supervisors/managers consider staff suggestions for improving patient safety, praise staff for following patient safety procedures, and do not overlook patient safety problems	4

11. Teamwork across units	Hospital units cooperate and coordinate with one another to provide the best care for patients	4

12. Teamwork within units	Staff support one another, treat each other with respect, and work together as a team	4

The survey also includes two outcome questions that ask respondents to provide an overall grade on patient safety for their work area/unit (A-Excellent, B-Very Good, C-Acceptable, D-Poor, E-Failing) and to indicate the number of events they have reported over the past 12 months (No events, 1 to 2 events, 3 to 5 events, 6 to 10 events, 11 to 20 events, or 21 events or more). In addition, respondents are asked to provide limited background demographic information about themselves (their work area/unit, staff position, whether they have direct interaction with patients, etc).

### Analyses

The goal of our analysis was to assess the psychometric properties of the AHRQ *Hospital Survey on Patient Safety *by verifying whether the 12 patient safety culture dimensions or composites existed and operated similarly at the individual, unit, and hospital levels of analysis. Descriptive statistics were produced to examine response variability and missing data.

#### Individual Level Factor Analysis

An individual level factor analysis was conducted to initially examine whether groups of items intended to measure a patient safety composite were interrelated, ignoring the nesting of data within units and within hospitals. Individual level factor analyses were conducted by specifying one factor for each a priori patient safety composite and then examining the factor loadings for each item in the composite. For an item to be considered as having an adequate contribution to a particular composite or factor, the strength of the item's relationship to that factor (i.e., its factor loading), should be .40 or greater [24].

Another statistic examined to determine the adequacy of a factor is the percent of variance accounted for by the factor. The more variance that is accounted for by a factor, the more justifiable it is to combine the items into a single composite score. The rule of thumb is that at least 50% of the variance should be accounted for by the composite.

#### Confirmatory Factor Analysis

Individuals responding to the Hospital SOPS are located within departments or units within hospitals. When data are nested in groups like this, results from an individual-level factor analysis may be biased or incorrect. Multilevel modelling may be more appropriate and necessary to account for the multilevel nature of the data. Therefore, multilevel confirmatory factor analysis was conducted on the a priori composites to examine the structure of the factors at the hospital and unit levels of analysis, taking into consideration that the data are nested.

#### Intraclass Correlations (ICCs) and Design Effects

To help determine the effect of nesting on the results, and to determine if multilevel analyses were necessary, intraclass correlations (ICCs) were computed for each composite using MPlus Version 5.1 [25]. ICC's determine if substantial variation exists between groups compared to variation within groups. ICCs above .05 or 5% indicate that the between group variance is greater than expected by chance and imply that nesting in groups does have an effect on the responses of individuals. Therefore, multilevel modelling would be necessary.

Given that ICCs are likely to be inflated when there are many groups with few individuals within the groups (compared to few groups with many individuals within the groups), we also examined design effects, which take into account within-group sample size (Design Effect = 1 + [Average within group sample size - 1] * ICC). A design effect of 2 or more implies that group membership or nesting of individuals within groups does have an effect on the responses of the individuals and therefore multilevel modelling should be conducted to account for the multilevel nature of the data.

#### Multilevel Confirmatory Factor Analyses (MCFA)

MCFAs were conducted using MPlus Version 5.1 to test the fit of measurement models for the 12 patient safety composites, taking into consideration the nested nature of the data. Two sets of multilevel confirmatory factor analyses were performed, one examining the unit level of analysis and a second examining the hospital level of analysis. The multilevel factor structure of the patient safety culture composites was tested examining each composite separately. We first evaluated the MCFA results by examining the item factor loadings on the composites when calculated at the unit level and then again at the hospital level. The rule of thumb is the same as for individual level factor analyses--that factor loadings at all levels should be .40 or greater.

Overall model fit statistics can only be computed for composites with four or more items. Therefore, we examined overall model fit indices using standard fit statistics: the chi-square, comparative fit index (CFI), and the standardized root mean square residual (SRMR) for six of the 12 composites that had four items. For the six composites with only three items, only factor loadings were used to assess the fit of the items.

For chi-square statistics, lower and non-significant chi-squares indicate good fit. Chi-square, however, is a function of sample size such that the larger the sample size the more likely it is that the chi-square will be significant. A large chi-square may emerge even when the model fits the data well; therefore two other model fit statistics were also examined: the CFI and SRMR. The CFI compares the existing model fit with a null model that assumes the items in the model are uncorrelated. The factor structure is determined to adequately fit the data if the CFI is at least .90 [26]. The standardized root mean square residual (SRMR) is the standardized difference between the observed covariance and predicted covariance. A value of zero for the SRMR indicates perfect fit, but a value less than .08 is considered a good fit [27].

#### Reliability Analysis

Reliability analyses were then performed on the final composites to ensure that individuals were responding consistently to the items within each composite. Internal consistency reliability was examined by calculating Cronbach's alpha for each of the composites to assess the extent to which respondents answered consistently to the theoretically similar items in each composite. Cronbach's alpha (α) ranges from 0 to 1.00, with higher alphas indicating better reliability. The minimum criterion for acceptable reliability is an alpha of at least .70 [28].

#### Intercorrelations

Intercorrelations among the patient safety composites and with the two outcome questions (Number of Events Reported and Patient Safety Grade) were also examined. Intercorrelations were explored at three levels of analysis: individual, unit, and hospital. While the composites should be correlated since they measure aspects of the patient safety culture, the intercorrelations should not be extremely high because very high intercorrelations indicate that the composites may not be unique enough to be considered separate constructs or measures. While there is no steadfast criterion about the magnitude of dimension intercorrelations and construct validity, in general, such correlations should be less than .80 for the composites to be considered unique and avoid problems with multicollinearity [29].

## Results

This section describes the results of the psychometric analysis of the AHRQ *Hospital Survey on Patient Safety*. Results are presented for each of the analytic steps described previously.

### Descriptive Statistics

The means, standard deviations, and percent positive scores for the survey items are provided in Additional File 1. All items showed good response variability (i.e., no items were found to have 90% or greater "agreement"--percentages of respondents answering positively) and there were low rates of missing data (ranging from 1% to 8% missing responses per item).

### Individual Level Factor Analysis

As shown in Additional File 2, all items within the composites had factor loadings above the .40 criterion, with an average loading of .80, and ranging from .59 to .92. The percent of variance accounted for by the composites in the survey (in parentheses in the second column in Additional File 2 on the row with the composite title) was above 50% for all but one composite, with an average of 64%; ranging from 47% to 77%. Staffing was the only composite that fell slightly below the 50% rule of thumb, at 47%. Overall, the individual level factor analysis results provided initial support for the 12 composites and justification for aggregation to a single composite score for each dimension. The next step investigated the composites, taking into account the nested nature of the data.

### Multilevel Analyses - ICCs and Design Effects

#### Unit-Level Analyses

As shown in Additional File 2, the item ICCs for the unit level were all above the .05 or 5% criterion (average ICC of .10; ranging from .06 to .23), indicating that between 6% and 23% of the variance in individual responses to the items could be attributed to department or unit membership. The design effects for the unit level were also all above the 2.00 criterion (average design effect of 3.10; ranging from 2.19 to 5.89). These two statistics confirmed that unit membership impacted the way individuals were responding to the survey, therefore the multilevel nature of the data needed to be taken into account when examining the factor structure for all 12 patient safety composites at the unit level.

#### Hospital-Level Analyses

ICC's and design effects were also calculated at the hospital level of analysis since individuals are also grouped or nested within hospitals. As shown in Additional File 2, several of the item ICCs for the hospital level fell below the .05 or 5% criterion (average ICC of .05; ranging from .02 to .10), indicating that between 2% and 10% of the variance in the individual items could be attributed to hospital membership. Because some of the low ICC values may have been low due to large within-hospital sample size (average number of respondents was 289 within hospitals), we examined design effects. The design effects at the hospital level all exceeded the 2.00 criterion (average design effect of 8.04; ranging from 3.99 to 16.15). Therefore, these two statistics confirmed that hospital membership also impacted the way individuals responded to the survey, and that the multilevel nature of the data needed to be taken into account when examining the factor structure for all 12 patient safety composites at the hospital level.

### Multilevel Analyses - Multilevel Confirmatory Factor Analysis (MCFA)

#### Unit-Level MCFA

At the unit level, the between-unit factor loadings ranged from .54 to 1.00 while the within-unit factor loadings ranged from .36 to .93 (see Additional File 2). One item, A7, "We use more agency/temporary staff than is best for patient care," in the Staffing composite had a low within-unit factor loading (.36). For the six composites with four items, overall model fit indices were also examined and are shown in Table 5. As seen in Table 5, chi-square tests for all six composites were significant (ideally a *non*-significant chi-square indicates good fit). However, the chi-square test is a rough estimate of fit and will frequently be significant due to a large sample size even if the model provides good fit to the data. Therefore, additional overall model fit indices were examined. Five of the six composites shown in Table 5 had comparative fit indices (CFIs) above the .90 criterion, with the exception of Supervisor/Manager Expectations & Actions Promoting Patient Safety (CFI = .88). In addition, the within- and between-unit standardized root mean square residuals (SRMRs) for all six composites were at or below the cutoff of .08 signifying good model fit. The within- and between-unit SRMR scores ranged from .01 to .07 for the within-unit models, and .01 to .06 for the between-unit models, indicating good model fit.

**Table 5:** Fit indices for Multilevel Analyses

	Unit Level					Hospital Level				
Hospital Survey Composites	χ^2^	df	CFI	Within SRMR	Between SRMR	χ^2^	df	CFI	Within SRMR	Between SRMR
Handoffs & Transitions	740.59*	5	.98	.02	.04	746.96*	5	.98	.02	.02
Overall Perceptions of Patient Safety	129.18*	4	1.00	.01	.01	143.56*	4	.99	.01	.04
Staffing	805.25*	4	.94	.04	.01	549.78*	4	.95	.04	.02
Supervisor/Manager Expectations & Actions Promoting Patient Safety	4731.02*	4	.88	.07	.06	5967.35*	4	.82	.07	.08
Teamwork Across Hospital Units	282.37*	4	.99	.02	.03	299.92*	4	.99	.01	.05
Teamwork Within Units	915.93*	5	.98	.02	.03	840.27*	4	.97	.03	.02

*Significant at p < .05

**Model fit indices can only be generated for factors with 4 or more items, therefore 6 composites were omitted from this table.

#### Hospital-Level MCFA

The between-hospital factor loadings ranged from .60 to 1.00 and the within-hospital factor loadings ranged from .36 to .93 (see Additional File 2). Similar to the unit-level results, item A7, "We use more agency/temporary staff than is best for patient care", in the Staffing composite, had a low within-hospital factor loading (.36). For the six composites with four items, overall model fit indices were also examined and are shown in Table 5. As seen in Table 5, chi-square tests for all six composites were significant (ideally a *non*-significant chi-square indicates good fit). However, five of the six composites shown in Table 5 had comparative fit indices (CFIs) above the .90 criterion, with the exception of Supervisor/Manager Expectations & Actions Promoting Patient Safety (CFI = .82). In addition, the within- and between-hospital SRMRs for all six composites were at or below the cutoff of .08 signifying good model fit. The within- and between-hospital SRMR scores ranged from .01 to .07 for the within-hospital models and .02 to .08 for the between-hospital models.

### Reliability Analysis

The reliability of the composites is shown in Table 6. Cronbach's alpha for the composites ranged from .62 to .85, with an average of .77. All composites had acceptable reliability (.70 or greater) except the Staffing composite (α = .62).

**Table 6:** Patient Safety Composite Reliability

Patient Safety Composite	Cronbach's Alpha Reliability
Communication openness	.73

Feedback & communication about error	.78

Frequency of event reporting	.85

Handoffs & transitions	.81

Management support for patient safety	.79

Nonpunitive response to error	.78

Organizational learning--Continuous improvement	.71

Overall perceptions of patient safety	.74

Staffing	.62

Supervisor/Manager expectations & actions promoting patient safety	.79

Teamwork across units	.79

Teamwork within units	.83

### Interrelations Among the 12 Patient Safety Culture Composites

Table 7 displays intercorrelations among the patient safety composites at the individual, unit, and hospital levels of analysis. The general pattern shows higher intercorrelations at higher levels of analysis: hospital higher than unit, and unit higher than individual-level correlations. Individual-level correlations averaged .42 (range: .19 to .64); unit-level correlations averaged .50 (range: .25 to .71); and hospital-level correlations averaged .56 (range: .31 to .81). The lowest intercorrelations at the individual, unit, and hospital levels were between Staffing and Frequency of Event Reporting (.19, .25, and .31 respectively). The highest intercorrelations at the individual, unit, and hospital levels were between Teamwork Across Units, and Handoffs and Transitions (.64, 71, and .81 respectively).

**Table 7:** Intercorrelations of Hospital SOPS Composites and Patient Safety Grades at the Individual, Unit, and Hospital Levels of Analysis

HSOPS Composite	COM- MUN	ER FREQ	FEED	HAND- OFF	MGMT	NON PUN	ORG LRN	OVER- ALL	STAFF	SUPV	TEAM-AC	TEAM- IN	GRADE
Frequency of event reporting (ERFREQ)													

Individual	.36	1											

Unit	.40	1											

Hospital	.52	1											

Feedback & communication about error (FEED)													

Individual	.62	.45	1										

Unit	.70	.52	1										

Hospital	.72	.65	1										

Handoffs & transitions (HANDOFF)													

Individual	.34	.28	.34	1									

Unit	.29	.31	.32	1									

Hospital	.35	.41	.42	1									

Management support for patient safety (MGMT)													

Individual	.44	.35	.50	.46	1								

Unit	.46	.40	.57	.50	1								

Hospital	.50	.53	.67	.58	1								

Nonpunitive response to error (NONPUN)													

Individual	.49	.23	.37	.33	.37	1							

Unit	.64	.32	.52	.35	.48	1							

Hospital	.61	.42	.51	.55	.51	1							

Organizational learning--Continuous improvement (ORGLRN)													

Individual	.48	.36	.58	.30	.54	.36	1						

Unit	.56	.47	.70	.31	.62	.48	1						

Hospital	.56	.58	.73	.32	.67	.39	1						

Overall perceptions of patient safety (OVERALL)													

Individual	.47	.37	.50	.45	.60	.44	.54	1					

Unit	.58	.42	.63	.46	.69	.60	.64	1					

Hospital	.64	.55	.70	.62	.75	.67	.64	1					

Staffing (STAFF)													

Individual	.34	.19	.31	.37	.42	.43	.33	.56	1				

Unit	.39	.25	.43	.45	.52	.50	.43	.67	1				

Hospital	.42	.31	.46	.62	.56	.58	.38	.76	1				

Supervisor/Mgr expectations & actions promoting patient safety (SUPV)													

Individual	.57	.33	.57	.32	.51	.45	.54	.53	.40	1			

Unit	.67	.42	.70	.34	.57	.60	.65	.62	.45	1			

Hospital	.67	.56	.75	.38	.65	.55	.68	.70	.48	1			

Teamwork across units (TEAMAC)													

Individual	.38	.28	.39	.64	.55	.33	.39	.46	.34	.37	1		

Unit	.37	.33	.43	.71	.63	.43	.44	.52	.45	.41	1		

Hospital	.39	.41	.50	.81	.69	.52	.51	.64	.57	.48	1		

Teamwork within units (TEAMIN)													

Individual	.49	.27	.44	.33	.39	.38	.51	.46	.36	.46	.41	1	

Unit	.61	.36	.56	.37	.42	.53	.61	.60	.43	.57	.45	1	

Hospital	.62	.46	.60	.47	.53	.54	.71	.69	.49	.62	.61	1	

Patient safety grade (GRADE)													

Individual	.49	.38	.52	.40	.57	.37	.53	.66	.43	.52	.43	.49	1

Unit	.56	.40	.59	.39	.60	.51	.58	.73	.53	.57	.46	.56	1

Hospital	.56	.43	.59	.41	.57	.51	.54	.69	.52	.58	.49	.57	1

Number of Events Reported (Events)													

Individual	.02	.003^ns^	.07	.12	.10	.003^ns^	.02	.14	.04	.03	.10	.03	.12

Unit	.04	.09	.05	.14	.11	.06	.03 ^ns^	.17	.10	.02 ^ns^	.07	.04 ^ns^	.15

Hospital	.09 ^ns^	.06 ^ns^	.13	.15	.06 ^ns^	.08 ^ns^	.01 ^ns^	.14	.12	.06 ^ns^	.07 ^ns^	.05 ^ns^	.16

^ns ^Signifies correlations that are **not **significant at p < .05.

The relationships between the patient safety composites and the two outcome items on the survey (Patient Safety Grade and Number of Events Reported) were also explored to determine if the composites were related to these self-reported outcome variables (see Table 7). For Patient Safety Grade, intercorrelations with the patient safety culture composites at the individual, unit, and hospital levels were all statistically significant. Individual-level correlations averaged .48 (range: .37 to .66); unit-level correlations averaged .55 (range: .39 to .73); and hospital-level correlations averaged .54 (range: .41 to .69). The highest intercorrelations at the individual, unit, and hospital levels were between Patient Safety Grade and Overall Perceptions of Patient Safety (.66, .73, and .69 respectively).

For Number of Events Reported, fewer of the intercorrelations with the 12 patient safety culture composites were statistically significant, particularly at the hospital level of analysis, and the magnitude of the significant relationships was low. The 10 individual-level correlations average .07 (range: .02 to .14); the nine unit-level correlations averaged .09 (range: .04 to .17); and the four hospital-level correlations averaged .14 (range:.12 to .15).

## Discussion

Overall, the results from the psychometric analyses--intraclass correlations (ICCs), design effects, MCFA results, model fit indices, item factor loadings, internal consistency reliability analyses, and dimension intercorrelations--all provide solid evidence supporting the 12 dimensions and 42 items included in the AHRQ Hospital Survey on Patient Safety Culture as having acceptable psychometric properties at the individual, unit and hospital levels of analysis, with a few exceptions. Our multilevel psychometric results indicate that both unit and hospital membership influence how individuals respond on the survey. The findings support our conclusion that the survey measures what it is supposed to: group culture at these higher levels, not just individual attitudes.

The Staffing composite fell slightly below cutoffs in a number of areas. Individual level factor analyses found that the percent of variance accounted for by Staffing fell slightly below the 50% rule of thumb, at 47%. In addition, one item in the Staffing composite had low within-unit and within-hospital factor loadings (.36--just below the .40 cutoff); the unit-level model fit was just below the .90 cutoff (CFI = .88); and the overall composite had low reliability (.62--below the .70 cutoff). Despite these findings, we recommend that the Staffing composite and items be retained due to the importance of staffing as emphasized in the 2003 Institute of Medicine report [30]. In addition, problems with staffing are often identified as a major theme of written comments on the survey. The factor analysis and reliability results did not point to any item in the Staffing composite that if dropped would improve the psychometric properties of the composite, which also indicates that the composite cannot be improved by dropping any of its three items.

The only other composite with a problematic psychometric finding was Supervisor/Manager Expectations & Actions Promoting Patient Safety in which the hospital-level model fit was lower than the cutoff of .90 (CFI = .82). Given that all other psychometrics for this scale were good, and its conceptual importance to patient safety, we also recommend retaining this composite.

The strongest relationships among the patient safety culture dimensions were between Overall Perceptions of Patient Safety and Patient Safety Grade and Management Support for Patient Safety. These strong correlations attest to the construct validity of the Overall Perceptions of Patient Safety composite. The findings also point to the important role hospital management plays in achieving patient safety [31] since staff rated their units higher on Patient Safety Grade when they perceived that hospital management supported patient safety.

Surprisingly, the weakest relationship was between Nonpunitive Response to Error and Frequency of Event Reporting. The existence of a nonpunitive culture appears to be only moderately associated with perceptions of event reporting. The strongest relationship with event reporting was with Feedback and Communication About Error, which highlights the importance of open communication about error and giving feedback about changes put into place based on event reports as potential means for increasing event reporting.

The one-item measure of the number of events staff reported in the past 12 months was disappointingly not related to any of the patient safety culture dimensions, perhaps due to the fact that our descriptive analysis discovered that 46% of staff had reported no events in the past year. For now, rather than using this as an outcome variable, perhaps it is best used as a descriptive measure to assess changes in staff event reporting over time until event reporting becomes more of a norm for staff in hospitals.

A strength of the survey is that it assesses a number of key cultural dimensions related to patient safety, focused at both the unit/department level, as well as hospital-wide. This multi-dimensional approach provides a level of specificity that makes it useful as a tool to guide patient safety improvement interventions. The results from the survey can be used to diagnose the current status of patient safety culture; raise staff awareness about patient safety; evaluate the impact of patient safety interventions and programs; trend culture change over time; conduct benchmarking with other hospitals; and fulfill regulatory directives and requirements [14].

It is also important to keep in mind that a quantitative survey is only one method that can be used to assess patient safety culture. Qualitative approaches involving observation, focus groups and interviews can provide more in-depth analysis and understanding of underlying cultural values and deeper cultural assumptions to complement data obtained from quantitative culture surveys. Additional methodological approaches can also be used to identify patient safety vulnerabilities, such as medical record review; patient safety indicators [32]; use of trigger tools to identify and quantify patient harm [33]; use of data from event reporting systems; root cause analysis; failure mode and effects analysis (FMEA); and probabilistic risk assessment [34].

Given widespread international interest in patient safety, the World Health Organization (WHO) is undertaking a multi-year High 5 s Project http://www.who.int/patientsafety/solutions/high5s/en/index.html to achieve reductions in high risk patient safety problems. Hospitals in participating countries have implemented the AHRQ Hospital SOPS to assess baseline patient safety culture and will track culture change over time as the initiative progresses. In addition, the European Network for Patient Safety (EUNetPaS-- http://90plan.ovh.net/~extranetn/) aims to establish an umbrella network of European Union Member States and stakeholders to encourage and enhance collaboration in the field of patient safety. One of the EUNetPaS key goals is to promote a culture of patient safety.

With the AHRQ Hospital SOPS translated into 18 languages and administered in over 30 countries, it is clear that there is a need for patient safety culture assessment tools around the world. A number of researchers that have administered the AHRQ Hospital SOPS in different countries have published psychometric results [35-38]. Analyses conducted by Smits et al (2009) in the Netherlands found strong psychometric support for 11 dimensions, with considerable unit-level variation. It is hoped that this proliferation of the survey's use and testing will result in a greater understanding of patient safety culture internationally as well as shed light on how to conduct cross-cultural comparisons on the survey results.

The ultimate goal of patient safety efforts is to reduce the risk of health care associated injury or harm to patients. A limitation of this study is that we were unable to examine the relationship between patient safety culture survey scores and indicators of actual patient harm either at the unit or hospital levels. Evidence about the criterion-related validity of patient safety culture instruments is much needed to examine the nature of the relationship between patient safety culture and patient outcomes. While there is abundant theory, case studies, and descriptive research on culture and culture change, there is still very little criterion-related research that links culture to "hard," non-perceptual outcomes like patient harm or cost savings. These are the data that move boards-of-directors and administrators to allocate resources and take action and are critical to telling the story of how patient safety culture impacts the bottom line.

More research is also needed about how to change culture. Hospitals that plan to implement patient safety culture interventions should work together with health services researchers to design rigorous studies of their interventions. Such collaborative research can produce evidence of the efficacy of cultural interventions that can be shared among hospitals interested in applying proven methods to guide how to change their patient safety culture in areas that need improvement.

## Conclusions

This study determined that the patient safety culture dimensions and items included in the *AHRQ Hospital Survey on Patient Safety Culture *are overall psychometrically sound for use by researchers and hospitals interested is assessing patient safety culture at the individual, unit and hospital levels. Further research is needed to study the criterion-related validity of the survey by analysing the relationship between patient safety culture and patient outcomes and studying how to improve patient safety culture. It is hoped that researchers and hospitals will use the survey tool assessed in this study to begin to shed light on the answers to some of these remaining questions about patient safety culture.

## Abbreviations

(AHRQ): The Agency for Healthcare Research and Quality; (CFI): Comparative fit index; (EUNetPaS): European Network for Patient Safety; (FMEA): Failure mode and effects analysis; (ICC): Intraclass correlation; (MCFA): Multilevel confirmatory factor analysis; (SRMR): Standardized root square mean residual; (SOPS): Survey on Patient Safety Culture.

## Competing interests

The authors declare that they have no competing interests.

## Authors' contributions

The authors contributed equally to this study and read and approved the final manuscript. ND performed the statistical analyses of the data. Both authors were involved with the interpretation of the data, and writing of the manuscript. Both authors read and approved the final manuscript.

## Pre-publication history

The pre-publication history for this paper can be accessed here:

http://www.biomedcentral.com/1472-6963/10/199/prepub

## Supplementary Material

Additional file 1 **AHRQ Hospital Survey on Patient Safety Culture Item Descriptive Statistics**. A table of means, standard deviations, and percent positive scores for each survey item. Click here for file

Additional file 2 **Factor Analysis and Reliability Results**. A table providing individual, unit and hospital level factor analysis results (factor loadings, percent of variance accounted for, ICC, design effect, within and between factor loadings). Click here for file
