# Supplementary material for: Multilevel psychometric properties of the AHRQ hospital survey on patient safety culture
Source: BMC Health Serv Res. 2010 Jul 8;10:199. doi: 10.1186/1472-6963-10-199 (PMC2912897; doi:10.1186/1472-6963-10-199)
Supplement: Additional file 1 — AHRQ Hospital Survey on Patient Safety Culture Item Descriptive Statistics. A table of means, standard deviations, and percent positive scores for each survey item. [file 1472-6963-10-199-S1.DOCX]

**Additional File 1. AHRQ Hospital Survey on Patient Safety Culture Item Descriptive Statistics**

| **Composite and Survey Item** | | **Mean** | **Std Dev** | **% Positive** |
| --- | --- | --- | --- | --- |
|  | **Communication Openness** |  |  |  |
| C2 | Staff will freely speak up if they see something that may negatively affect patient care. | 3.95 | 0.85 | 75% |
| C4 | Staff feel free to question the decisions or actions of those with more authority. | 3.27 | 1.07 | 45% |
| C6R | Staff are afraid to ask questions when something does not seem right. | 3.67 | 0.96 | 62% |
|  | **Feedback and Communication about Error** |  |  |  |
| C1 | We are given feedback about changes put into place based on event reports. | 3.45 | 0.99 | 51% |
| C3 | We are informed about errors that happen in this unit. | 3.66 | 1.01 | 60% |
| C5 | In this unit, we discuss ways to prevent errors from happening again. | 3.80 | 0.95 | 67% |
|  | **Frequency of Events Reporting** |  |  |  |
| D1 | When a mistake is made, but is caught and corrected before affecting the patient, how often is this reported? | 3.38 | 1.10 | 48% |
| D2 | When a mistake is made, but has no potential to harm the patient, how often is this reported? | 3.49 | 1.08 | 52% |
| D3 | When a mistake is made that could harm the patient, but does not, how often is this reported? | 3.96 | 0.99 | 71% |
|  | **Handoffs & Transitions** |  |  |  |
| F3R | Things “fall between the cracks” when transferring patients from one unit to another. | 2.94 | 1.02 | 34% |
| F5R | Important patient care information is often lost during shift changes. | 3.27 | 1.00 | 48% |
| F7R | Problems often occur in the exchange of information across hospital units. | 3.04 | 0.96 | 37% |
| F11R | Shift changes are problematic for patients in this hospital. | 3.15 | 1.01 | 41% |
|  | **Management Support for Patient Safety** |  |  |  |
| F1 | Hospital mgmt provides a work climate that promotes patient safety. | 3.80 | 0.91 | 76% |
| F8 | The actions of hospital mgmt show that patient safety is a top priority. | 3.69 | 0.98 | 68% |
| F9R | Hospital mgmt seems interested in patient safety only after an adverse event happens. | 3.31 | 1.10 | 54% |
|  | **Nonpunitive Response to Error** |  |  |  |
| A8R | Staff feel like their mistakes are held against them. | 3.23 | 1.08 | 47% |
| A12R | When an event is reported, it feels like the person is being written up, not the problem. | 3.10 | 1.08 | 42% |
| A16R | Staff worry that mistakes they make are kept in their personnel file. | 2.88 | 1.04 | 31% |
|  | **Organizational Learning - Continuous Improvement** |  |  |  |
| A6 | We are actively doing things to improve patient safety. | 3.96 | 0.82 | 81% |
| A9 | Mistakes have led to positive changes here. | 3.54 | 0.89 | 60% |
| A13 | After we make changes to improve patient safety, we evaluate their effectiveness. | 3.63 | 0.85 | 66% |

Note: The item number is shown in the first column. An “R” indicates items that are negatively worded and reverse-scored when calculating percent positive scores.

**Additional File 1. AHRQ Hospital Survey on Patient Safety Culture Item Descriptive Statistics (page 2)**

| **Composite and Survey Item** | | **Mean** | **Std Dev** | **% Positive** |
| --- | --- | --- | --- | --- |
|  | **Overall Perceptions of Safety** |  |  |  |
| A10R | It is just by chance that more serious mistakes don’t happen around here. | 3.46 | 1.14 | 58% |
| A15 | Patient safety is never sacrificed to get more work done. | 3.43 | 1.13 | 58% |
| A17R | We have patient safety problems in this unit. | 3.42 | 1.10 | 57% |
| A18 | Our procedures and systems are good at preventing errors from happening. | 3.64 | 0.90 | 68% |
|  | **Staffing** |  |  |  |
| A2 | We have enough staff to handle the workload. | 3.09 | 1.22 | 48% |
| A5R | Staff in this unit work longer hours than is best for patient care | 3.31 | 1.08 | 52% |
| A7R | We use more agency/ temporary staff than is best for patient care. | 3.85 | 1.05 | 67% |
| A14R | We work in “crisis mode” trying to do too much, too quickly. | 3.06 | 1.11 | 44% |
|  | **Supervisor/manager Expectations & Actions Promoting Safety** |  |  |  |
| B1 | My supv/mgr says a good word when he/she sees a job done according to established patient safety procedures. | 3.68 | 1.07 | 68% |
| B2 | My supv/mgr seriously considers staff suggestions for improving patient safety. | 3.80 | 1.00 | 74% |
| B3R | Whenever pressure builds up, my supv/mgr wants us to work faster, even if it means taking shortcuts. | 3.77 | 0.98 | 72% |
| B4R | My supv/mgr overlooks patient safety problems that happen over and over. | 3.91 | 1.03 | 75% |
|  | **Teamwork Across Hospital Units** |  |  |  |
| F2R | Hospital units do not coordinate well with each other. | 2.94 | 1.07 | 37% |
| F4 | There is good cooperation among hospital units that need to work together. | 3.31 | 0.97 | 50% |
| F6R | It is often unpleasant to work with staff from other hospital units. | 3.37 | 0.98 | 54% |
| F10 | Hospital units work well together to provide the best care for patients. | 3.55 | 0.92 | 60% |
|  | **Teamwork Within Hospital Units** |  |  |  |
| A1 | People support one another in this unit. | 4.01 | 0.93 | 83% |
| A3 | When a lot of work needs to be done quickly, we work together as a team to get the work done. | 4.06 | 0.89 | 84% |
| A4 | In this unit, people treat each other with respect. | 3.80 | 0.98 | 74% |
| A11 | When one area in this unit gets really busy, others help out. | 3.55 | 1.08 | 65% |

Note: The item number is shown in the first column. An “R” indicates items that are negatively worded and reverse-scored when calculating percent positive scores.
