# Supplementary material for: Multilevel psychometric properties of the AHRQ hospital survey on patient safety culture
Source: BMC Health Serv Res. 2010 Jul 8;10:199. doi: 10.1186/1472-6963-10-199 (PMC2912897; doi:10.1186/1472-6963-10-199)
Supplement: Additional file 2 — Factor Analysis and Reliability Results. A table providing individual, unit and hospital level factor analysis results (factor loadings, percent of variance accounted for, ICC, design effect, within and between factor loadings). [file 1472-6963-10-199-S2.DOC]

## Additional File 2. Factor Analysis and Reliability Results

| **Hospital Survey**  **Composites and Items** | | **Indiv**  **Level Factor Analysis** | **Multilevel Factor Analysis** | | | | | | | |
| --- | --- | --- | --- | --- | --- | --- | --- | --- | --- | --- |
|  |  |  | **_______Unit Level_________** | | | | **______Hospital Level_______** | | | |
|  |  | **Factor Loading**  **(% var accounted)** | **ICC** | **Design Effect** | **Within Factor Load-ing** | **Between Factor Loading** | **ICC** | **Design Effect** | **Within Factor Load-ing** | **Between Factor Loading** |
| **Communication Openness** | | **(65%)** |  |  |  |  |  |  |  |  |
| C2 | Staff will freely speak up if they see something that may negatively affect patient care. | 0.81 | .07 | 2.47 | .68 | .92 | .03 | 5.48 | .69 | .91 |
| C4 | Staff feel free to question the decisions or actions of those with more authority. | 0.83 | .07 | 2.47 | .73 | .93 | .03 | 5.48 | .74 | .90 |
| C6R | Staff are afraid to ask questions when something does not seem right. | 0.78 | .06 | 2.26 | .60 | .97 | .02 | 3.99 | .62 | .95 |
| **Feedback and Communication about Error** | | **(70%)** |  |  |  |  |  |  |  |  |
| C1 | We are given feedback about changes put into place based on event reports. | 0.83 | .09 | 2.89 | .70 | .86 | .04 | 6.97 | .71 | .82 |
| C3 | We are informed about errors that happen in this unit. | 0.83 | .08 | 2.68 | .71 | .87 | .03 | 5.48 | .72 | .88 |
| C5 | In this unit, we discuss ways to prevent errors from happening again. | 0.85 | .10 | 3.09 | .76 | .99 | .04 | 6.97 | .77 | .99 |
| **Frequency of Events Reported** | | **(77%)** |  |  |  |  |  |  |  |  |
| D1 | When a mistake is made, but is caught and corrected before affecting the patient, how often is this reported? | 0.86 | .07 | 2.39 | .74 | .90 | .03 | 5.24 | .75 | .90 |
| D2 | When a mistake is made, but has no potential to harm the patient, how often is this reported? | 0.92 | .07 | 2.39 | .93 | .99 | .03 | 5.24 | .93 | .98 |
| D3 | When a mistake is made that could harm the patient, but does not, how often is this reported? | 0.86 | .06 | 2.19 | .73 | .92 | .03 | 5.24 | .74 | .88 |
| **Handoffs & Transitions** | | **(63%)** |  |  |  |  |  |  |  |  |
| F3R | Things “fall between the cracks” when transferring patients from one unit to another. | 0.79 | .12 | 3.47 | .69 | .95 | .08 | 12.73 | .70 | .97 |
| F5R | Important patient care information is often lost during shift changes. | 0.81 | .07 | 2.44 | .71 | .91 | .04 | 6.86 | .72 | .94 |
| F7R | Problems often occur in the exchange of information across hospital units. | 0.82 | .08 | 2.64 | .75 | .99 | .05 | 8.33 | .76 | .99 |
| F11R | Shift changes are problematic for patients in this hospital. | 0.75 | .09 | 2.85 | .62 | .82 | .06 | 9.80 | .62 | .95 |

## Additional File 2. Factor Analysis and Reliability Results (Page 2)

|  | | **Indiv**  **Level Factor Analysis** | **Multilevel Factor Analysis** | | | | | | | |
| --- | --- | --- | --- | --- | --- | --- | --- | --- | --- | --- |
|  |  |  | **________Unit Level________** | | | | **______Hospital Level______** | | | |
| **Hospital Survey**  **Composites and Items** | | **Factor Loading**  **(% var accounted)** | **ICC** | **Design Effect** | **Within Factor Load-ing** | **Between Factor Loading** | **ICC** | **Design Effect** | **Within Factor Load-ing** | **Between Factor Loading** |
| **Mgmt Support for Patient Safety** | | **(71%)** |  |  |  |  |  |  |  |  |
| F1 | Hospital mgmt provides a work climate that promotes patient safety. | 0.84 | .11 | 3.30 | .71 | .96 | .06 | 9.95 | .73 | .92 |
| F8 | The actions of hospital mgmt show that patient safety is a top priority. | 0.88 | .11 | 3.30 | .86 | .99 | .05 | 8.46 | .87 | .98 |
| F9R | Hospital mgmt seems interested in patient safety only after an adverse event happens. | 0.80 | .08 | 2.67 | .62 | .92 | .05 | 8.46 | .63 | .87 |
| **Nonpunitive Response to Error** | | **(69%)** |  |  |  |  |  |  |  |  |
| A8R | Staff feel like their mistakes are held against them. | 0.84 | .10 | 3.12 | .72 | .99 | .04 | 7.03 | .74 | .99 |
| A12R | When an event is reported, it feels like the person is being written up, not the problem. | 0.84 | .10 | 3.12 | .74 | .95 | .04 | 7.03 | .75 | .95 |
| A16R | Staff worry that mistakes they make are kept in their personnel file. | 0.82 | .10 | 3.12 | .67 | .96 | .05 | 8.54 | .69 | .96 |
| **Org Learning- Continuous Improvement** | | **(63%)** |  |  |  |  |  |  |  |  |
| A6 | We are actively doing things to improve patient safety. | 0.81 | .11 | 3.33 | .69 | .99 | .06 | 10.07 | .70 | .98 |
| A9 | Mistakes have led to positive changes here. | 0.77 | .08 | 2.70 | .59 | .88 | .04 | 7.04 | .60 | .90 |
| A13 | After we make changes to improve patient safety, we evaluate their effectiveness. | 0.80 | .09 | 2.91 | .66 | .92 | .05 | 8.56 | .67 | .95 |
| **Overall Perceptions of Patient Safety** | | **(57%)** |  |  |  |  |  |  |  |  |
| A10R | It is just by chance that more serious mistakes don’t happen around here. | 0.75 | .12 | 3.54 | .60 | .93 | .05 | 8.55 | .63 | .96 |
| A15 | Patient safety is never sacrificed to get more work done. | 0.74 | .10 | 3.12 | .58 | .89 | .05 | 8.55 | .60 | .89 |
| A17R | We have patient safety problems in this unit. | 0.79 | .15 | 4.18 | .69 | .98 | .06 | 10.06 | .71 | .96 |
| A18 | Our procedures and systems are good at preventing errors from happening. | 0.74 | .10 | 3.12 | .58 | .94 | .04 | 7.04 | .61 | .74 |

## Additional File 2. Factor Analysis and Reliability Results (Page 3)

|  | | **Indiv**  **Level Factor Analysis** | **Multilevel Factor Analysis** | | | | | | | |
| --- | --- | --- | --- | --- | --- | --- | --- | --- | --- | --- |
|  |  |  | **_______Unit Level_______** | | | | **_______Hospital Level______** | | | |
| **Hospital Survey**  **Composites and Items** | | **Factor Loading**  **(% var accounted)** | **ICC** | **Design Effect** | **Within Factor Load-ing** | **Between Factor Loading** | **ICC** | **Design Effect** | **Within Factor Load-ing** | **Between Factor Loading** |
| **Staffing** | | **(47%)** |  |  |  |  |  |  |  |  |
| A2 | We have enough staff to handle the workload. | 0.72 | .23 | 5.89 | .54 | .89 | .10 | 16.15 | .58 | .88 |
| A5R | Staff in this unit work longer hours than is best for patient care | 0.66 | .08 | 2.70 | .44 | .72 | .03 | 5.54 | .44 | .84 |
| A7R | We use more agency/ temporary staff than is best for patient care. | 0.59 | .16 | 4.40 | .36 | .54 | .08 | 13.12 | .36 | .60 |
| A14R | We work in “crisis mode” trying to do too much, too quickly. | 0.77 | .15 | 4.19 | .67 | .94 | .08 | 13.12 | .70 | .92 |
| **Suprv/Manager Expectations & Actions Promoting Patient Safety** | | **(61%)** |  |  |  |  |  |  |  |  |
| B1 | My supv/mgr says a good word when he/she sees a job done according to established patient safety procedures. | 0.81 | .10 | 3.09 | .78 | .94 | .04 | 6.96 | .79 | .94 |
| B2 | My supv/mgr seriously considers staff suggestions for improving patient safety. | 0.85 | .11 | 3.30 | .86 | 1.00 | .04 | 6.96 | .87 | .99 |
| B3R | Whenever pressure builds up, my supv/mgr wants us to work faster, even if it means taking shortcuts. | 0.76 | .09 | 2.88 | .54 | .81 | .04 | 6.96 | .56 | .77 |
| B4R | My supv/mgr overlooks patient safety problems that happen over and over. | 0.70 | .07 | 2.46 | .47 | .90 | .03 | 5.47 | .49 | .89 |
| **Teamwork Across Hospital Units** | | **(62%)** |  |  |  |  |  |  |  |  |
| F2R | Hospital units do not coordinate well with each other. | 0.79 | .09 | 2.87 | .67 | .91 | .06 | 9.89 | .69 | .86 |
| F4 | There is good cooperation among hospital units that need to work together. | 0.82 | .09 | 2.87 | .74 | .99 | .05 | 8.40 | .76 | 1.00 |
| F6R | It is often unpleasant to work with staff from other hospital units. | 0.71 | .07 | 2.45 | .55 | .84 | .04 | 6.92 | .56 | .86 |
| F10 | Hospital units work well together to provide the best care for patients. | 0.82 | .09 | 2.87 | .74 | .97 | .06 | 9.89 | .75 | .98 |
| **Teamwork Within Units** | | **(66%)** |  |  |  |  |  |  |  |  |
| A1 | People support one another in this unit. | 0.86 | .13 | 3.77 | .83 | 1.00 | .05 | 8.58 | .85 | .97 |
| A3 | When a lot of work needs to be done quickly, we work together as a team to get the work done. | 0.83 | .11 | 3.34 | .73 | .90 | .04 | 7.06 | .74 | .95 |
| A4 | In this unit, people treat each other with respect. | 0.84 | .14 | 3.98 | .78 | .96 | .05 | 8.58 | .79 | .97 |
| A11 | When one area in this unit gets really busy, others help out. | 0.71 | .11 | 3.34 | .54 | .80 | .04 | 7.06 | .55 | .90 |
